# Long-Term Impact of Intralesional Bony Overgrowth on Opposing Cartilage Integrity: Five-Year Results Following Cartilage Repair

**DOI:** 10.1177/19476035251335008

**Published:** 2025-04-28

**Authors:** Felix Ragnar Merlin Koenig, Marcus Raudner, Gregor Wollner, Vladimir Juras, Pavol Szomolanyi, Veronica Vetchy, Johannes Leitner, Victor Schmidbauer, Siegfried Trattnig

**Affiliations:** 1 High-Field MR Center, Department of Biomedical Imaging and Image Guided Therapy, Medical University of Vienna, Vienna, Austria; 2 Department of Biomedical Imaging and Image Guided Therapy, Medical University of Vienna, Vienna, Austria; 3 Department of Orthopedics and Trauma-Surgery, Medical University of Vienna, Vienna, Austria; 4 Institute of Measurement Science, Slovak Academy of Sciences, Bratislava, Slovakia; 5 Austrian Cluster for Tissue Regeneration, Vienna, Austria; 6 Institute for Clinical Molecular MRI in the Musculoskeletal System, Karl Landsteiner Institute for Clinical Molecular MR, Vienna, Austria

**Keywords:** knee joint, articular cartilage, subchondral arthroplasty, bone formation, magnetic resonance imaging, follow-up studies

## Abstract

**Objectives:**

This study aimed to assess the impact of intralesional bony overgrowth (ILBO) after cartilage repair on the integrity of opposing articulating cartilage (OpAC) using T2 mapping and to correlate these findings with clinical outcomes.

**Methods:**

In this multicenter study, magnetic resonance imaging (MRI) examinations were performed in the follow-up after cartilage repair (Microfracturing (MFX) and Matrix-Induced Autologous Chondrocyte Implantation (MACI)) in 45 patients up to 5 years after surgery. T2 values of the OpAC after 3, 12, and 60 months in patients with and without ILBO after 60 months were conducted along with clinical assessments (International Knee Documentation Committee (IKDC) and Knee injury and Osteoarthritis Outcome Score (KOOS)).

**Results:**

At 60 months post-surgery, 44.4% of patients presented with ILBO, which was associated with significantly higher T2 values in OpAC (*P* = 0.004). A tendency toward increased T2 values was observed after 12 months, although this did not reach statistical significance (*P* = 0.06). However, no significant differences were found in clinical outcomes between patients with or without ILBO, nor between those with or without T2 values comparable to reference cartilage.

**Conclusion:**

ILBO significantly affects the biophysical MRI properties of OpAC as indicated by higher T2 values after 60 months. These alterations, though not reflected in any clinical score, can suggest potential long-term implications for cartilage degeneration and may inform future monitoring strategies for cartilage repair. Further research is required to evaluate the long-term effects of these altered mechanical impacts on articulating cartilage and their clinical implications.

## Key Points

- The composition of opposing articulating cartilage is significantly influenced by intralesional bony overgrowth after cartilage repair.
- MRI T2 mapping shows significant changes in the composition of opposing articulating cartilage 60 months after cartilage repair surgery.
- No impact on clinical outcome could be detected after 60 months.

## Introduction

Magnetic resonance imaging (MRI) is the method of choice for morphological imaging of cartilage and cartilage repair; quantitative magnetic resonance (MR) methods, such as T2 mapping, offer a noninvasive method of identifying early degenerative alterations within cartilage^1,2^ and can monitor the cartilage composition and maturation after cartilage repair.^3^ Therefore, the integration of compositional imaging through T2 mapping is beneficial for the evaluation of cartilage repair tissue and its incorporation with the surrounding native tissues.^1^ T2 mapping is widely available, easy to implement, and does not require special hardware on the MR scanner. Studies with histological correlation have already reported higher T2 values in osteoarthritis (OA),^4^ as well as substantiated findings regarding the prospective utility of T2 mapping to quantify and predict the progression of cartilage defects in untreated cases.^2^

The articular cartilage, subchondral lamina, and subchondral bone are increasingly recognized as a functional unit, playing a significant role in overall joint health.^5^ While the formation of conventional osteophytes has been extensively studied and linked to factors such as aging, trauma, and mechanical stress,^6^ the phenomenon of intralesional bony overgrowth (ILBO), which can arise following cartilage repair, remains relatively unexplored in a systematic manner. The underlying mechanisms driving ILBO are still not well understood.^7^ The aim of this study was to assess the impact of ILBO on the integrity of opposing articulating cartilage (OpAC) using T2 mapping as a non-invasive marker. Studies have shown that ILBO increases the stiffness of the cartilage surface, creating a more rigid environment that places greater mechanical stress on the opposing cartilage during joint movement.^8^ Additionally, ILBO reduces the cartilage’s natural shock absorption capacity, transferring more of the mechanical load to the opposing tissue.^9^ The presence of ILBO may also alter joint biomechanics, leading to abnormal loading patterns that further affect the opposing cartilage.^10^

The objective of this study is to evaluate how ILBO affects the biophysical MRI properties of OpAC, specifically by examining T2 values, and to correlate these findings with clinical outcomes. Identifying early biophysical MRI changes before clinical symptoms become evident may offer the opportunity for improved monitoring strategies and more targeted interventions, ultimately enhancing patient management and preserving joint function over time.

## Methods

### Study Design and Inclusion Criteria

This retrospective data analysis involved patients who participated in a multicenter, prospective, randomized controlled, and open-label study. Participants were informed in detail about the participation and gave written informed consent. The study was done in accordance with the Declaration of Helsinki and was approved by the respective ethics committees and regulatory authorities at each participating site.

Enrollment criteria required that patients have a localized articular cartilage defect of the femoral condyle or the trochlea of the knee, with a defect grade of III or IV as per the International Cartilage Repair Society (ICRS) classification and a defect size between 2 and 6 cm^2^. Patients had an intact meniscus, with up to 50% resection permitted. In the absence of any medical history data, the condition of the meniscus might be assumed at Visit 1. Knee joint are stable or ligaments have been sufficiently repaired; if not, ligament repair needs to be done before to, during, or no later than 6 weeks after cartilage treatment. Patients exhibited unrestricted movement in the afflicted knee joint or experienced a maximum deviation of 10° in extension and flexion. The exclusion criteria included the following: inability to undergo specific medical imaging, previous cartilage repair surgeries, degenerative joint diseases with a Kellgren and Lawrence grade of 2 or higher, inflammatory arthritis, malalignment (specifically valgus- or varus deformity), systemic diseases, infections, a body mass index higher than 35 kg/m², substance abuse, cognitive impairments, active systemic or local microbial infection at the surgery site, and a known history of cancer within the past 5 years.

**Figure 1.**
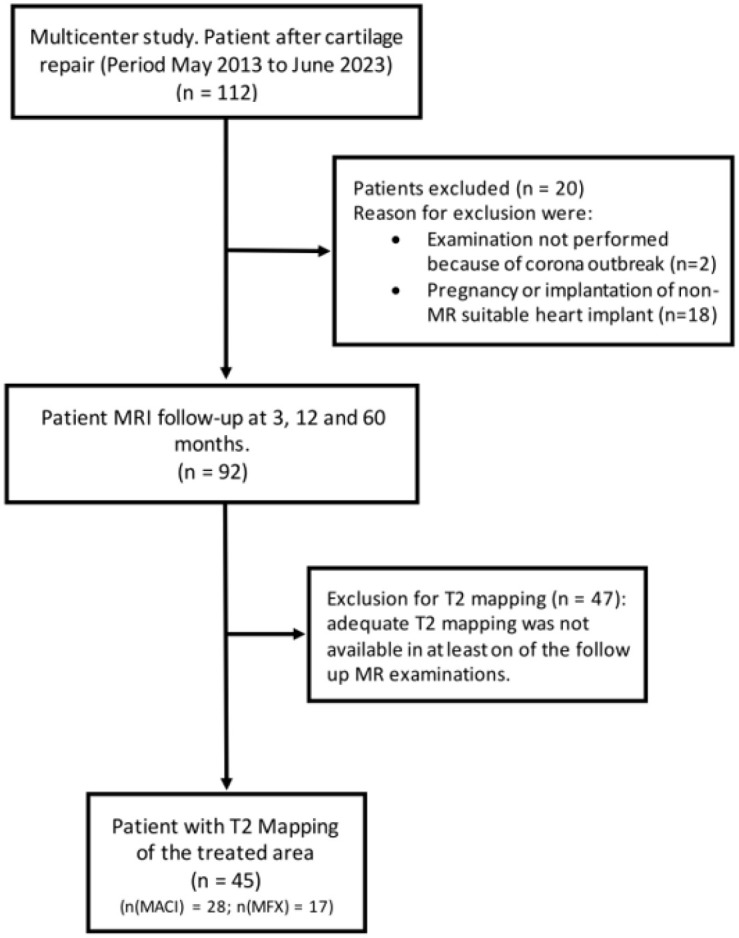
Flowchart of this retrospective study.

A total of 45 patients from this MRI study with adequate T2 maps and MRI examinations available at all follow-up visits (3, 12, and 60 months after cartilage repair) were included in the present analysis. Of these, 28 (62.2%) were patients of the Matrix-Induced Autologous Chondrocyte Implantation (MACI) group, and 17 (37.5%) were from the Microfracturing (MFX) group. The flowchart of this retrospective study is shown in **Figure 1**.

### MR Examination

All examinations for this study were performed on a 3 Tesla whole-body MR scanner. MRI scans were conducted using Siemens 3T MRI machines (Siemens Healthineers, Erlangen, Germany), Philips Medical Systems 3T MRI machines (Philips Medical Systems, Best, Netherlands), and a GE Medical Systems 3T MRI machine (GE Medical Systems, Chicago, IL, USA). For this multicenter study, vendor-specific multi-element knee coils, ranging from 8 to 16 coils, were used. The comprehensive MR examination protocol and its corresponding sequence parameters are presented in detail in **Table 1** as already described in earlier studies.^11^

**Table 1.** Parameters of the MRI Protocol Used in This Trial [9].

Parameter/sequence	T2 Mapping	TSE PD	TSE T2w	SE T1w
Orientation plane	Sagittal	Coronal	Sagittal	Sagittal
Averages	1	2	3	1
Flip angle (°)	90,180	180	180	90
Acquisition matrix	320 × 256	448 × 403	381 × 448	448 × 381
Image matrix	320 × 320	896 × 896	448 × 448	448 × 448
Field of view (cm)	16 × 16	16 × 16	16 ×16	16 × 16
Slice thickness (mm)	3	3	2	2
Slice spacing (mm)	3.3	3.3	2.2	2.2
Repetition time (ms)	2000	3080	3310	700
Echo time (ms)	12.5; 25; 37.5; 50; 62.5; 75; 87.5; 100	28	83	12
Total acquisition time (min: sec)	10:36	04:46	03:55	03:53

The study protocol comprised a morphological component, which included the Turbo Spin Echo (TSE) Proton Density (PD), TSE T2 weighted (w), and SE T1w sequences, and a compositional component with T2 mapping that used a multi-echo multi-slice sequence. The acquisition of T2 maps was carried out with the parameters listed in **Table 1**.

The primary reading site received all images, used a two-parametric exponential fitting method to generate T2 maps, and conducted segmentation and grading for cartilage restoration.

### Image Analysis

**Figure 2.**
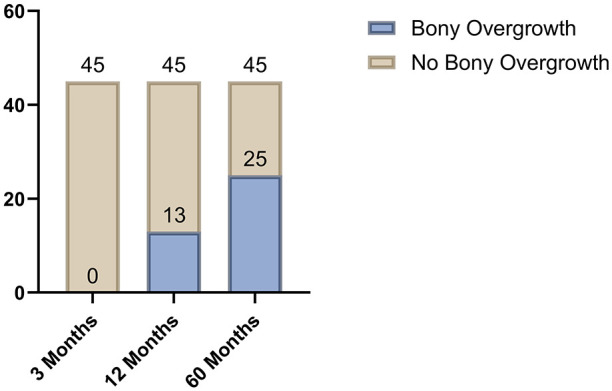
Appearance of ILBO 3 to 60 months after surgery: 55.6% of all patients presented with bony overgrowth after 60 months.

The T2 values were evaluated using ITK SNAP^12^ by two radiologists with over 6 years of experience in MSK MRI. A minimum of three ROIs were drawn in the OpAC directly opposite the cartilage repair tissue in a minimum of three contiguous sagittal sections. For individual reference of the T2 values, tibial morphologically inconspicuous, homogenous cartilage with a minimum distance of 1 cm from the OpAC was identified, and three ROIs in at least three contiguous sagittal sections were drawn (see **Fig. 2**). The same orientation relative to B0 than the articulating graft tissue was chosen, to exclude magic angle effect.

The mean of the T2 values of the OpAC was divided by the mean of the T2 values of the morphologically inconspicuous tibial cartilage. The resulting global articulating T2 ratio was used for statistical analyses. The reproducibility of T2 mapping has already been demonstrated in several other studies.^13^

The semiquantitative evaluation of the morphological outcome following a cartilage repair procedure was carried out using the MOCART 2.0 (Magnetic Resonance Observation of Cartilage Repair Tissue 2.0).^14^ This system was designed to systematically document the status of the cartilage repair area and surrounding tissues. Its reliability, reproducibility, and applicability across different surgical cartilage repair techniques have been well-demonstrated.^15,16^

This scoring system comprises seven variables, each contributing to a maximum achievable score of 100. The variables include: MOCART 2.0 system subscores, including the volume fill of the cartilage defect (0-20 points); integration into the adjacent cartilage (0-15 points); surface of the repair tissue (0-10 points); structure of the repair tissue (0-10 points); signal intensity of the repair tissue (0-10 points); bony defect or bony overgrowth (0-10 points); and subchondral changes (0-20 points).^14^

ILBO was assessed on fat-suppressed PD MRI sequences in the coronal plane. According to the MOCART Score 2.0, cartilage repair with intact subchondral bone was rated as “no bony overgrowth”; ILBO was subcategorized as bony overgrowth <50% and ≥50% of the thickness of adjacent native cartilage.^14^

The evaluations were conducted by an experienced senior musculoskeletal radiologist, who has over 30 years of experience in the field. They were performed on MR examinations 3, 12, and 60 months after cartilage repair.

### Clinical Scores

To assess clinical progress in this study, patient-reported outcomes were used. Specifically, the Knee injury and Osteoarthritis Outcome Score (KOOS) and the subjective International Knee Documentation Committee (IKDC) score were used as clinical correlation measures. These assessments were conducted separately by each site in this multicenter study following general recommendations.^17,18^

The KOOS is a self-reported questionnaire that evaluates five subscales of knee function: pain, symptoms, daily living activities, sport and recreation, and quality of life. The IKDC, on the other hand, is a clinician-administered questionnaire that assesses overall knee function. Both scores were implemented according to standard guidelines. Evaluations were performed at baseline (prior to intervention) and at 3, 12, and 60 months post-intervention.

### Statistical Evaluation

The statistical analysis of the data was conducted by a biomedical statistician using IBM SPSS version 24.0.1 for Windows (IBM, Chicago, IL). To examine the data, several statistical tests were employed. Chi-square tests were used to analyze the association between categorical variables. The Mann-Whitney *U* test was used to compare differences between two independent groups for continuous or ordinal variables that were not normally distributed. The Kruskal-Wallis test (Posthoc test: Dunn’s test) was applied to compare more than two independent groups. Tests for normal distribution were conducted to determine the suitability of these methods (Shapiro-Wilk test). These statistical analyses provided a thorough examination of the data.

Images were generated with GraphPad Prism version 10 (GraphPad Software, La Jolla California USA, www.graphpad.com) and SPSS. A *P* value of 0.05 or less was considered statistically significant.

## Results

This retrospective data analysis included 45: Thirteen females and 32 males with a mean age of 40.1 ± 10.3 years at surgery. After 60 months, 7 women (53%) and 13 men (41%) had no ILBO, ILBO was detected by 6 women (46%) and 19 men (59%). Chi-square tests showed no significant associations between ILBO and sex (*P* = 0.42). Average age at surgery was 40.09 ± 10.25. Mann-Whitney *U* test was conducted to compare the age at intervention between those with and without ILBO, with a *P* value of 0.61, indicating no significant difference regarding age between the two groups.

At the 3-month follow-up, none of the patients in either the MACI or MFX groups exhibited ILBO. After 12 months, a total of 13 patients (29%) in the combined cohort demonstrated bony overgrowth: In nine lesions (20%), the extent of ILBO was <50% of the thickness of adjacent native cartilage, and in four lesions (9%), the extent was ≥50%. At the 60-month evaluation, a total of 25 patients (56%) presented with bony overgrowth, 14 (31%) of those with an extent <50% of the thickness of adjacent native cartilage, 11 (24%) with an extent of ILBO ≥50%. In contrast, 20 patients (44%) remained free from ILBO after 60 months.

### T2 Mapping

**Figure 3.**
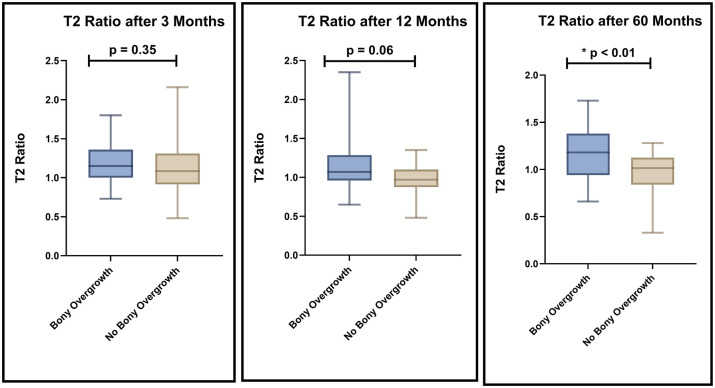
T2 ratio of the opposing articulating cartilage in patient with ILBO (n = 25) and without ILBO (n = 20) after 60 months: No differences after 3 months, a tendency but no significant difference after 12 months and significant difference between the ILBO and No ILBO group after 60 months.

Good interrater agreement (kappa > 0.80) was found between the two radiologists who evaluated the T2 values of the OpAC at 3, 12, and 60 months. In addition, intraclass correlation (ICC) was good (ICC > 0.85) at these time points. The global T2 ratio of the tibial OpAC was compared between patients with ILBO and those without ILBO (n = 20) at 60 months. The T2 ratio was similar between groups at 3 months (median 1.15 (ILBO) vs 1.08 (no ILBO), *P* = 0.36). At 12 months, there was already a non-significant trend toward a difference between the groups (median 1.07 vs 0.97, *P* = 0.06). At 60 months, a statistically significant difference in OpAC T2 ratios was observed (median 1.18 vs. 1.02, *P* < 0.01). The Hodges-Lehmann estimate, a robust measure of the median difference, was -0.22, further confirming the statistical significance of the difference in T2 values at 60 months between the two groups. The following box plots (**Fig. 3**) visualize the result.

**Figure 4.**
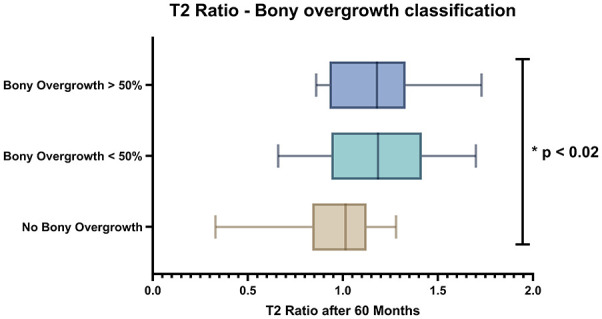
T2 ratio after 60 months in patient with no ILBO (n = 20), with minor ILBO (n= 14) and major ILBO (n = 11): Significant difference in both minor bony overgrowth (<50%) and major bony overgrowth (>50%) compared to the no ILBO group.

Statistical significance was also seen between the OpAC T2 ratio after 60 months across different bony overgrowth classifications (*P* < 0.02). The analysis involved three groups (no bony overgrowth (n = 20), bony overgrowth <50 (n = 14), and bony overgrowth ≥50% (n = 11)), illustrated in **Figure 4**. These differences were significant for both groups of bony overgrowth compared with no bony overgrowth (no bony overgrowth and bony overgrowth <50%; *P* < 0.02; no bony overgrowth and bony overgrowth ≥50%: *P* = 0.02).

**Figure 5.**
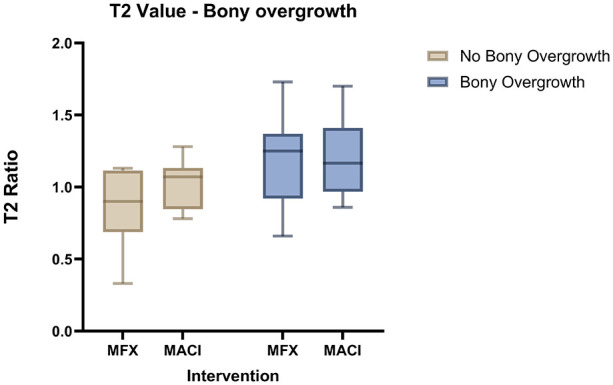
Differences in ILBO in the treatment groups: No ILBO: MFX vs. MACI, median 0.90 (IQR 0.4) vs. 1.07 (IQR: 0.3); ILBO: MFX vs. MACI, median 1.25 (IQR: 0.5) vs. 1.16 (IQR: 0.4).

With consideration to the different treatment approaches, 20 patients in which no ILBO was detected, 8 patients were treated with MFX and 12 patients were treated with MACI. ILBO were detected in 9 patients in the MFX group and 16 patients in the MACI group. Two-way analysis of variance (ANOVA; post-hoc test Hochberg GT2) showed no significant differences between the groups and the T2 OpAC ratio (*P* = 0.43), as shown in **Figure 5**. However, due to the small sample size, the respective medians were compared (MFX: no ILBO/ILBO, median 0.90 vs. 1.25, interquartile range [IQR] 0.4 vs. 0.5; MACI: no ILBO/ILBO, median: 1.07 vs. 1.16, IQR 0.3 vs. 0.4).

**Figure 6.**
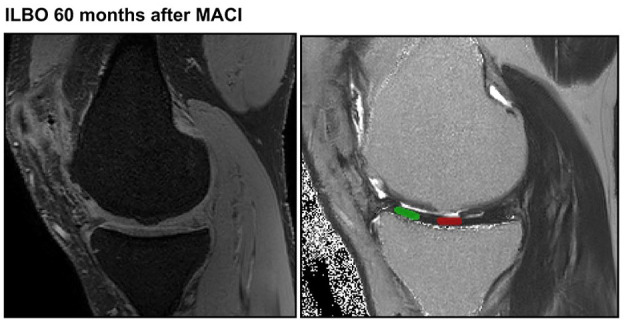
ILBO after 60 months following MACI: After 3 months, no bony overgrowth was detectable. There were differences in the T2 values of the articulating cartilage (red) and the reference cartilage of the tibia (green).

Reference image with ILBO at 60 months and T2 measurements is provided in **Figure 6**.

### MOCART Score

The mean MOCART 2.0 Score increased from 65.68 ± 19.93 at 3 months, to 74.09 ± 20.36 at 12 months, and to 76.59 ± 13.019 at 60 months. There were no statistically significant differences in the MOCART 2.0 scores between the MFX and MACI group (3 months: *P* = 0.09, 12 months: *P* = 0.75, 60 months: *P* = 0.76).

### Clinical Scores

Regarding the clinical scores, the Kruskal-Wallis test was conducted to assess differences in KOOS and IKDC scores across three groups based on the degree of bony overgrowth (No Bony Overgrowth, Bony Overgrowth <50%, and Bony Overgrowth >50%) at 3, 12, and 60 months post-intervention.

The results of the Kruskal-Wallis test revealed no statistically significant differences among the groups for KOOS at 3 months (*P* = 0.35), 12 months (*P* = 0.99), and 60 months (*P* = 0.57). Similarly, no significant differences were found in IKDC scores at 3 months (*P* = 0.20), 12 months (*P* = 0.92), and 60 months (*P* = 0.88).

We categorized the T2 ratio of OpAC at 60 months as “similar to tibial reference cartilage” (T2 ratio of OpAC greater than 0.8 and less than 1.2) and “not similar to reference cartilage” (T2 ratio of OpAC less than 0.8 and greater than 1.2). We compared these two groups in terms of IKDC and KOOS scores, including their subscales, at 60 months. KOOS and IKDC Dynamic were defined as the change in score from baseline (immediately after the intervention) to 60 months.

The results showed no statistically significant differences between the two groups in the IKDC and KOOS scores, including all subscales. See **Table 2** for more details.

**Table 2.** Comparison of IKDC and KOOS Scores, Including Their Subscales, Between T2 Ratios Categorized in Similar (0.8 > x < 1.2, n = 28) and Not Similar (0.8 < x > 1.2, n = 17) to Tibial Reference Cartilage at 60 Months.

Scores	T2 Ratio of OpAC Similar to Tibial Reference Cartilage	T2 Ratio of OpAC Not Similar to Tibial Reference Cartilage	*P* Value
IKDC 60 months	22.0	24.65	0.51
IKDC Dynamic	21.61	25.29	0.36
KOOS 60 months	22.84	23.26	0.92
KOOS Dynamic	21.43	25.59	0.30
Symptoms KOOS	24.38	20.74	0.37
Function Daily KOOS	23.21	22.65	0.89
Function Sport KOOS	22.91	23.15	0.95
Quality of Life KOOS	22.11	24.47	0.56
Pain KOOS	23.32	22.47	0.83

Mean ranks are presented for each group, alongside the p-values obtained from the Mann-Whitney U test. No statistically significant differences were observed between the groups across all scores (*P* > 0.05).

## Discussion

The study aimed to assess the impact of ILBO on the biophysical MRI properties of the OpAC using T2 mapping. Our findings indicate that while ILBO leads to increased T2 values in the OpAC, suggesting early biophysical MRI changes, these alterations do not currently correlate with significant differences in clinical outcomes. This effect was significantly measurable by compositional MR with T2 mapping in the presented patient cohort of 45 individuals, 60 months after cartilage repair surgery. No MRI morphological correlation was seen on the fat-suppressed PD MRI sequences of the tibial cartilage. After 3 and 12 months, there were no significant differences in the T2 values of the OpAC, although there was a nonsignificant trend toward higher global T2 ratios in the OpAC with ILBO in cartilage repair present after 12 months. This can be explained by the slow development of bony overgrowth as a complication after cartilage repair surgery and the resulting different biomechanical effects on the OpAC.

In this multicenter study, we aimed to provide a quantitative assessment that is relatively independent of the MRI system hardware and MR sequence protocol. To achieve this, we calculated a global T2 ratio between the opposing area after cartilage repair and tibial reference cartilage of the same knee, a method previously used in several studies, including Krusche-Mandl *et al.*^19^

Lin *et al.*^4^ correlated T2 values of the femoral condyle of the knee with histological grading of OA and showed that T2 values can identify OA changes in cartilage. In a “normal” postoperative course, the T2 relaxation times of the repair tissue, whether through MFX or ACI (Autologous chondrocyte implantation) procedures, displayed an initial increase during the early postoperative months. Subsequently, these T2 values gradually decreased to levels below those seen in reference cartilage, as shown in a comprehensive review by Jungmann *et al.*^20^ Focusing on the articular cartilage in direct opposition to these treated lesions, a steadily rising T2 ratio in a nontreated area can be assumed to be early cartilage degeneration caused by the excessive bone formation in bony overgrowth and consecutive nonphysiological load profiles. The elevated T2 ratio of OpAC following ILBO development may predict new cartilage defects in the cartilage region opposite the repair site. Similar results were reported in a quite small study that followed 12 patients after cartilage repair, 25 years after surgery, which demonstrated a correlation between ILBO and cartilage damage in the opposite cartilage.^21^ It is important to mention that long-term follow-ups are indicated here, as, in our study, these changes only appeared after several years. Most of the studies on this topic refer only to a 2-year follow-up, in which these cartilage lesions may not be detectable.

Our results support the hypothesis that even small ILBO had a significant influence on the global T2 ratio of OpAC. As in the MOCART Score 2.0, we classified ILBO into bony overgrowth <50% and ≥50% of the thickness of adjacent native cartilage.^14^ Even smaller ILBO showed higher T2 ratios of the OpAC after 60 months, so it could be assumed that even slightly altered mechanical properties influence the OpAC. The disparity in how cartilage and bone adapt disturbs the vital interplay between these tissues, which is pivotal for upholding the regular structure and function of the joint and may play a role in advancing the progression of OA pathology.^22^ In a study by Knutsen *et al.*,^24^ both MFX and ACI were insufficient to prevent the progression of OA after 15 years. Our results show that ILBO may play a role in this development.

Our study population had a rate of ILBO formation of 56% after 60 months. There are not many comparable studies available, but the rate was lower compared to studies after more than 60 months, such as that published by Vasiliadis *et al.*^24^ with a reported incidence of bony overgrowth of 64% of extended follow-up cases (9-18 years) following ACI. The rates of occurrence of ILBO associated with cartilage repair have exhibited considerable variation, spanning from 25% to 70% in the case of bone marrow stimulation/microfracture procedures, and ranging from 23% to 64% for ACI.^24-27^ This emphasizes the frequency of ILBO after cartilage repair.

The observed elevation in T2 values aligns with previous research demonstrating that ILBO can increase the stiffness of the cartilage surface, thereby transferring more mechanical load to the opposing tissue.^8^ This increased load may reduce the cartilage’s natural shock absorption capacity and potentially lead to abnormal loading patterns, contributing to cartilage degeneration over time.^9,10^ It is important to underline that T2 mapping reflects water content and collagen organization and serves as a sensitive, albeit indirect, marker of cartilage matrix changes rather than a direct biochemical analysis.^2,3^ However, despite these biophysical MRI changes, our study did not find corresponding differences in clinical scores, which may be attributed to the sensitivity of patient-reported outcome measures in detecting early biophysical MRI alterations. Several studies have shown similar results regarding a discrepancy in clinical scores: Shive *et al.*^7^ reported no relationship between bony overgrowth and clinical outcome after 12 months, as did Niemeyer *et al.*^28^ in 10 years after ACI. Recently Zak *et al.*^10^ published a paper with a 77% ILBO rate after MACI and no significant changes in clinical outcomes at 60 to 120 months. This discrepancy may stem from clinical scores, which assess overall joint function rather than localized changes in cartilage. The biomechanical alterations and early degenerative changes in the OpAC might not be severe enough to produce clinical symptoms within the 60-month follow-up. In addition, the relatively young age and high functional baseline of our cohort may have helped compensate for biomechanical changes, delaying clinical manifestations. Since early OA due to focal cartilage degeneration can remain asymptomatic, a 5-year follow-up may not be sufficient to capture its long-term effects. Further studies with extended follow-up periods will be essential to better understand the potential development of early OA and its clinical implications over time.

Most studies have focused primarily on the repair tissue, rather than the OpAC. This narrow focus may overlook important mechanical changes that occur in the OpAC following ILBO after cartilage repair. Although these changes in T2 values did not correspond to differences in clinical scores at the 60-month follow-up, this discrepancy may result from the nature of clinical scores, which assess overall knee function rather than localized changes in cartilage properties. Therefore, MR-based morphological and biophysical assessments of both the treated area and surrounding cartilage are essential for a more comprehensive evaluation of cartilage repair outcomes. Our findings are consistent with previous studies suggesting that early biophysical MRI changes detected through MRI may precede visible clinical symptoms.^2^ These results highlight the value of advanced imaging techniques like T2 mapping in identifying early signs of cartilage degeneration, even when clinical measures appear unchanged. Our findings are consistent with studies that have employed T2 mapping MRI to evaluate cartilage quality and repair tissue. For instance, a study by Årøen *et al.*^29^ used MRI T2 mapping and delayed Gadolinium Enhanced MRI of Cartilage to assess degenerative changes in cartilage surrounding focal lesions, highlighting the utility of these imaging modalities in detecting early degenerative changes.

While it remains uncertain whether these changes become clinically significant after 10 years, their relevance may be heightened due to the younger age of patients undergoing cartilage repair (mean age in our study: 40 years; Zak et al.: 33 years). Further long-term studies are necessary to determine whether these changes progress to clinical symptoms. In summary, understanding the relationship between ILBO and T2 values is crucial and recognizing these early biophysical MRI changes can inform postoperative monitoring strategies, allowing clinicians to identify and address potential issues before they progress to clinically significant stages. This proactive approach can lead to more personalized and effective intervention strategies, ultimately improving patient outcomes and preserving joint function. Incorporating advanced imaging techniques, such as T2 mapping, into routine postoperative assessments can enhance the ability to monitor cartilage health and guide clinical decision-making.

In our study sample, there was no clear difference in ILBO between MFX and MACI, but caution must be taken because of the small sample size. As mentioned before, there is a lack of systematic evaluation of ILBO after cartilage repair.

This study has several limitations that should be considered when interpreting the results. First, the small sample size of 45 patients, although normal for other comparable studies, may limit the statistical power and the generalizability of the results. We acknowledge that using different MRI machines across centers may introduce variability in T2 mapping results. To minimize this, we standardized the imaging protocol and used the same multi-echo sequence. We also calculated a dimensionless T2 ratio, comparing affected opposing cartilage with reference tissue of the same patient, which reduces equipment-related variability and ensures comparability across different MRI systems. In addition, ILBO was classified into two broad categories based on the extent of overgrowth (<50% or ≥50%). While this classification provides a useful distinction, further granularity, such as using 25% intervals, could offer more precise insights into how varying degrees of ossification impact the OpAC. Finally, the 60-month follow-up period, while substantial, may be insufficient to detect the long-term clinical implications of the observed biophysical changes in OpAC.

It is crucial to emphasize that, particularly in the context of cartilage repair, morphological and MRI-detectable alterations do not necessarily correlate with functional impairment or clinical symptoms. While ILBO demonstrates consistent imaging characteristics, the lack of clinical impact over a substantial 5-year period can also support the interpretation of ILBO as primarily an imaging feature without immediate clinical consequence.

Nevertheless, our findings demonstrate that ILBO significantly influences the biophysical MRI properties of the OpAC, as evidenced by elevated T2 values suggestive of early cartilage alterations. However, these MRI-based changes did not translate into measurable differences in patient-reported clinical outcomes after 60 months. Considering the progressive nature of cartilage degeneration and OA, particularly in younger, active patient populations, extended follow-up remains essential to determine whether these imaging findings may eventually acquire clinical relevance.

Ongoing research should focus on the progression of these altered mechanical characteristics and their potential role in long-term joint health deterioration, ultimately aiming to clarify their contribution to OA development over extended periods.
